# Fatal pufferfish poisoning 1000 km inland: a case series from eastern Turkey

**DOI:** 10.2478/aiht-2026-77-4088

**Published:** 2026-06-30

**Authors:** Mehmet Tatlı, Selin Bulut

**Affiliations:** University of Health Sciences Van Training and Research Hospital, Emergency Medicine Department, Van, Turkey; Military Medical City Hospital, Emergency Medicine Department, Doha, Qatar

**Keywords:** food poisoning, *Lagocephalus sceleratus*, paralysis, refractory cardiac arrest, respiratory failure, tetrodotoxin, *Lagocephalus sceleratus*, paraliza, refraktorni srčani zastoj, respiratorno zatajenje, trovanje hranom, tetrodotoksin

## Abstract

We report the first tetrodotoxin (TTX) poisoning cases from Van, Eastern Turkey, a city located more than 1000 km inland. Three male patients presented in December 2020 after eating *Lagocephalus sceleratus*, which a local vendor mistook for an edible fish. One patient, a 41-year-old man who consumed the fish liver, rapidly developed severe signs of poisoning (adverse event grade 4) with complete paralysis and respiratory failure. Arterial blood gas analysis showed severe acidosis (pH 7.179) and high lactate levels (5.3 mmol/L). Despite intensive care, including mechanical ventilation and high-dose vasopressors, the patient died on the fourth day of hospitalisation due to refractory cardiac arrest. The other two patients, who consumed less fish and no liver, had only mild symptoms (adverse event grades 1 and 2) and recovered completely within 24 h. This incident illustrates the extreme danger of consuming pufferfish and particularly its visceral organs, which typically contain substantially higher tetrodotoxin levels. Finding these cases so far inland reveals serious gaps in the Turkish food distribution system. The fish likely travelled from Mediterranean ports to Van in mixed fish crates without having been identified. Fish vendors need proper training to identify toxic species, and supply chains require better oversight to prevent such tragedies.

## Abstract (hr)

Ovo je prvi prikaz niza slučajeva trovanja tetrodotoksinom (TTX) u gradu Vanu u istočnoj Turskoj, smještenom više od 1000 km od obale. Tri muškarca primljena su u hitnu službu u prosincu 2020. nakon konzumacije otrovne napuhače, vrste *Lagocephalus sceleratus*, koju je mjesni prodavač zamijenio s jestivom ribom. Jedan bolesnik, 41-godišnji muškarac koji je pojeo riblju jetru, ubrzo je dobio teške znakove trovanja (4. stupanj štetnih događaja) s potpunom paralizom i respiratornim zatajenjem. Analiza plinova arterijske krvi pokazala je tešku acidozu (pH 7,179) i povišene vrijednosti laktata (5,3 mmol/L). Unatoč intenzivnom liječenju, uključujući mehaničku ventilaciju i primjenu visokih doza vazopresora, bolesnik je preminuo četvrtoga dana hospitalizacije zbog refraktornoga srčanog zastoja. Druga dva bolesnika, koja su pojela manju količinu mesa ribe i nisu jela jetru, imali su samo blage simptome (1. i 2. stupanj štetnih događaja) te su se potpuno oporavili unutar 24 sata. Ovaj incident potvrđuje iznimnu opasnost konzumacije napuhača, osobito iznutrica, koje obično sadržavaju znatno više razine tetrodotoksina. Činjenica da je do trovanja došlo tako daleko od obale upozorava na ozbiljne propuste u turskom sustavu distribucije hrane. Riba je vjerojatno dopremljena iz mediteranskih luka u Van u sanducima s miješanom ribom, a da pritom nije bila identificirana. Prodavači ribe trebaju proći odgovarajuću edukaciju za prepoznavanje otrovnih vrsta, a opskrbni lanci morali bi imati bolji nadzor kako bi se spriječile ovakve tragedije.

Tetrodotoxin (TTX) poisoning is a severe toxicological emergency primarily caused by the consumption of pufferfish (family *Tetraodontidae*) (1). The silver-cheeked toadfish *(Lagocephalus sceleratus*), an invasive species from the Red Sea, has rapidly spread throughout the Mediterranean, including the Turkish coast. This expansion has led to an increase in poisoning cases in the region, where the local population is unfamiliar with the lethal nature of this species (2).

This report documents the first known TTX poisoning cases in the city of Van in eastern Turkey, which is over 1000 km inland from the Mediterranean coast.

## CASE REPORTS

This case series was prepared in accordance with the CARE Guidelines (3) and reviews the records of three patients who presented to the Emergency Department of the Van Training and Research Hospital in December 2020. Written informed consent for publication was obtained from the surviving patients and the next of kin of the deceased patient. The diagnosis was based on the patients' clinical findings and history, with the silver-cheeked toadfish species confirmed by fish images. Laboratory confirmation of TTX in the fish or the patients was not possible because we had no facilities in our region to do that in time.

In December 2020, three adult males in Van purchased fish mislabelled as 'iron fish' (a fabricated local name) from a local vendor lacking any training in fish species identification (in Turkish, *Demir Balığı* translates literally to 'iron fish', but no such species exists, as vendors in inland regions often invent names for unfamiliar marine fish). The three victims shared a single large specimen after cooking it in a stone oven. One ate the liver, deeming it a particularly nutritious delicacy, while the other two ate the muscle tissue.

Table 1 compares the clinical and laboratory characteristics of the three patients.

**Table 1:** Comparative clinical and laboratory characteristics of the three patients poisoned with pufferfish in Van, Turkey

**Parameter**	**Case 1 (Fatal)**	**Case 2 (Recovered)**	**Case 3 (Recovered)**
Age/Gender	41/Male	31/Male	41/Male
Symptom onset (min)	90	240	360
Fish part consumed	Liver (150–200 g)	Muscle (100 g)	Muscle (50 g)
Estimated TTX dose^*^	2.44 mg	~0.38 mg	~0.19 mg
Initial symptoms	Perioral numbness, dizziness, vomiting	Perioral numbness, malaise	Perioral numbness, fatigue
CTCAE grade	4	2	1
Blood pressure (mmHg)	70/40	110/75	120/80
Heart rate (bpm)	105	98	96
SpO_2_ (%)	80	94	95
pH	7.179	7.38	7.40
pCO_2_ (mmHg)	63.5	38	36
Lactate (mmol/L)	5.3	1.2	1.1
Mechanical ventilation	Yes	No	No
Vasopressor support	Yes (high-dose)	No	No
ICU admission	Yes	No	No
Hospitalisation (days)	4	1	1
Complications	Aspiration pneumonia, cerebral oedema	None	None
Outcome	Death (Day 4)	Full recovery	Full recovery

* based on consumed quantity and tissue concentrations reported by Christidis et al. (8); CTCAE – Common Terminology Criteria for Adverse Events (4)

### Case 1: fatal outcome

This 41-year-old man (body weight: 82 kg), previously healthy with no chronic diseases, consumed approximately 200 g of the fish liver. About 90 min after the meal, he began experiencing perioral numbness and tingling in his fingertips. Within minutes, the symptoms rapidly progressed. The patient developed severe dizziness, nausea, and profuse vomiting. His family noticed that he had difficulty speaking and walking.

When he arrived at our Emergency Department 30 min after symptom onset, his condition was already critical. He was in severe shock, with a blood pressure of 70/40 mmHg and a heart rate of 105 beats/min. His oxygen saturation was dangerously low, at 80 %, despite supplemental oxygen therapy. He rapidly progressed to flaccid paralysis affecting all four limbs. He could barely move his arms or legs, and his respiration was visibly weakening.

Initial arterial blood gas analysis revealed severe mixed acidosis (pH 7.179, pCO_2_ 63.5 mmHg) with significant hyperlactataemia (5.3 mmol/L), indicating profound tissue hypoperfusion. We immediately intubated the patient for impending respiratory failure and placed him on mechanical ventilation. Despite aggressive vasopressor support with high-dose norepinephrine (titrated up to >0.5 µg/kg/min), his blood pressure remained unstable.

Over the next four days in the intensive care unit, we attempted every possible intervention. We maintained mechanical ventilation, continued high-dose vasopressor therapy, corrected metabolic acidosis, and provided supportive care, but the patient's condition continued to deteriorate. He developed bilateral aspiration pneumonia and cerebral oedema. On the fourth hospital day, despite maximal resuscitative efforts, including cardiopulmonary resuscitation, the patient succumbed to refractory cardiorespiratory arrest.

### Case 2: moderate exposure, full recovery

The second victim, a 31-year-old man (body weight: 78 kg), consumed approximately 100 g of muscle tissue but avoided the liver. He first noticed symptoms about 4 h (240 min) after the meal, much later than Case 1. He experienced mild perioral numbness, a tingling sensation around the mouth, and general malaise. He felt weak but could still walk and talk normally.

Upon arrival at our Emergency Department, his vital signs were stable. His blood pressure was 110/75 mmHg, heart rate 98 beats/min, and oxygen saturation 94 % on room air. Neurological examination revealed only mild sensory changes around the mouth. The laboratory results were essentially normal. We admitted the patient for observation and provided supportive care with intravenous fluids.

His symptoms gradually improved over the next 12 h. By hour 24, the patient was completely asymptomatic. We discharged him with the advice to avoid pufferfish consumption. At the 6-month follow-up, he had no neurological sequelae and had returned to normal life.

### Case 3: minimal exposure, full recovery

The third victim, a 41-year-old male (body weight: 75 kg), consumed only a small amount of muscle tissue (approximately 50 g) and no liver. He developed symptoms even later, approximately 6 h (360 min) after the meal. His symptoms were the mildest among the three, with only slight perioral numbness and mild fatigue.

When he presented to our Emergency Department, his vital signs were normal. The blood pressure was 120/80 mmHg, heart rate 96 beats/min, and oxygen saturation 95 %. His physical examination was unremarkable, except for mild perioral paraesthesia. Laboratory tests revealed no abnormalities.

We kept the patient under observation for 24 h with supportive care. His symptoms resolved completely within 18 h. The patient was discharged and remained well at follow-up.

## DISCUSSION

To our knowledge, this is the first report of TTX poisoning in inland Turkey. These three cases clearly indicate the dose-dependent effects of this potent neurotoxin, considering that the patient who consumed the liver experienced grade 4 adverse events, according to the Common Terminology Criteria for Adverse Events (CTCAE) (4), and died, whereas the other two had grades 2 and 1, respectively, and recovered fully (Table 1). This stark difference demonstrates the critical importance of the consumed tissue type for clinical outcomes (5).

TTX blocks sodium channels in the nerves and muscles, preventing electrical signals from traveling through these tissues (1). This explains why patients develop paralysis. In our patients, we observed the typical progression: numbness around the mouth appeared first, followed by ascending paralysis and finally respiratory failure. This sequence reflects the effects of TTX on different nerve types. The deceased patient remained conscious until the late stages of the illness, because TTX cannot cross the blood-brain barrier (6).

We could not measure TTX levels in fish or patients due to the lack of laboratory facilities in our region. However, the diagnosis was straightforward based on the clinical picture and confirmed history of pufferfish consumption. In resource-limited settings such as ours, TTX poisoning is usually diagnosed based on clinical features and exposure history rather than laboratory confirmation (7).

Based on reported consumption amounts and average tissue-specific TTX concentrations reported by Christidis et al. (8), we estimated the exposure dose of 2.44 mg for Case 1 (consumption of ~200 g liver with mean TTX 12.2 µg/g), which exceeds the lethal threshold of 2 mg. In contrast, Cases 2 and 3 consumed muscle tissue with much lower TTX levels and estimated exposure doses.

Although no specific antidote exists, some experimental approaches such as anticholinesterase agents (e.g., neostigmine or edrophonium) have been suggested in the literature (9), though their efficacy remains unproven and management primarily relies on aggressive supportive care.

### Incident ramifications: a food safety crisis

**Figure 1:**
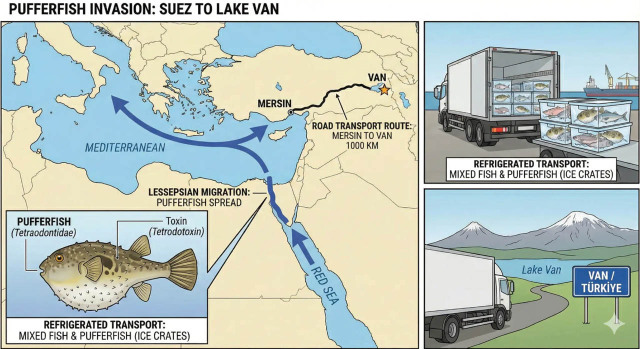
Pufferfish invasion route from the Suez Canal and likely supply route to the city of Van, Turkey

The described incident in Van, which is over 1000 km distant from the coast, points to a dangerous new pattern of TTX poisoning (Figure 1). Historically, this issue was confined to coastal areas where pufferfish are caught. It has now reached deep inland populations through unregulated food distribution networks.

*Lagocephalus sceleratus* originally entered the Mediterranean from the Red Sea through the Suez Canal (2). This invasive species has rapidly spread across the Eastern Mediterranean, including the Turkish coastal waters. It is now commonly caught in Mediterranean ports alongside other commercial fish species.

Fish are typically sorted by species at coastal ports, but inexperienced workers could inadvertently place the toxic pufferfish into crates containing edible species before transport. These mixed crates are then transported hundreds or even thousands of kilometres inland to cities such as Van. Fish travel in refrigerated trucks, passing through multiple distribution points, but no one checks for toxic species along this chain. When these mixed fish crates reach inland cities, they are handled by local vendors, who generally have no experience with marine species, but rely on the following dangerous assumption: "If it came from the sea, it must be edible." This cultural belief, common in inland regions, creates a deadly vulnerability.

The vendor who sold the fish in our case had no formal training in fish species identification. He had been selling fish for years, trusting that his suppliers would only send edible species. When he saw *L. sceleratus* in his shipment, he did not recognise it as being dangerous.

This lack of knowledge proved fatal and has exposed multiple system failures. First, Mediterranean ports of Turkey lack mandatory sorting procedures for removing toxic species before transport. Second, the fish distribution chain has no checkpoints for verifying species. Third, inland vendors do not receive training in identifying toxic marine species. Fourth, there are no public awareness campaigns warning inland populations about the dangers of pufferfish.

## CONCLUSION

Considering that there are no antidotes to TTX poisoning, safety measures should focus on prevention.

Several urgent interventions are required. Mediterranean ports must implement mandatory sorting procedures to remove pufferfishes before inland transport. Fish should be checked along the distribution chain for species verification. Fish vendors, especially those in inland regions, should have mandatory training to identify toxic fish species. Public awareness campaigns should warn the population about the dangers of pufferfish.

Emergency physicians in inland areas should also be made aware that TTX poisoning can occur far from the sea.
